# The B_sister_ MADS Gene *FST* Determines Ovule Patterning and Development of the Zygotic Embryo and Endosperm

**DOI:** 10.1371/journal.pone.0058748

**Published:** 2013-03-19

**Authors:** Dong Sun Lee, Li Juan Chen, Cheng Yun Li, Yongsheng Liu, Xue Lin Tan, Bao-Rong Lu, Juan Li, Shu Xian Gan, Sang Gu Kang, Hak Soo Suh, Youyong Zhu

**Affiliations:** 1 Key Lab of Agro-Biodiversity and Pest Management of Education Ministry, Yunnan Agricultural University, Kunming, China; 2 Rice Research Institute, Yunnan Agricultural University, Kunming, China; 3 Key Lab of Molecular Breeding for Dian-Type Japonica Hybrid Rice of Yunnan Education Department, Yunnan Agricultural University, Kunming, China; 4 Ministry of Education Key Lab for Bio-resource and Eco-environment, College of Life Science, State Key Lab of Hydraulics and Mountain River Engineering, Sichuan University, Chengdu, China; 5 Ministry of Education Key Lab for Biodiversity and Ecological Engineering, Institute of Biodiversity Science, Fudan University, Shanghai, China; 6 School of Biotechnology, Yeungnam University, Gyeongsan, Korea; 7 School of Biological Resources, Yeungnam University, Gyeongsan, Korea; Nanjing Agricultural University, China

## Abstract

Many homeotic MADS-box genes have been identified as controllers of the floral transition and floral development. However, information regarding B_sister_ (B_s_)-function genes in monocots is still limited. Here, we describe the functional characterization of a B_s_-group MADS-box gene *FEMALE-STERILE* (*FST*), whose frame-shift mutation (*fst*) results in abnormal ovules and the complete abortion of zygotic embryos and endosperms in rice. Anatomical analysis showed that the defective development in the *fst* mutant exclusively occurred in sporophytic tissues including integuments, fertilized proembryos and endosperms. Analyses of the spatio-temporal expression pattern revealed that the prominent *FST* gene products accumulated in the inner integument, nucellar cell of the micropylar side, apical and base of the proembryos and free endosperm nuclei. Microarray and gene ontology analysis unraveled substantial changes in the expression level of many genes in the *fst* mutant ovules and seeds, with a subset of genes involved in several developmental and hormonal pathways appearing to be down-regulated. Using both forward and reverse genetics approaches, we demonstrated that rice *FST* plays indispensable roles and multiple functions during ovule and early seed development. These findings support a novel function for the B_s_-group MADS-box genes in plants.

## Introduction

Sexual reproduction in higher plants includes a key phase to produce male and female gametes, ensuring pollination and fertilization. The female gametophyte is essential for the sexual reproduction of plants [1]–[3]. During the last few years, significant progress has been made in determining the molecular components that control ovule identity, embryo sac polarity, gametophytic cell specification, female gametic cell fate determination, embryogenesis and endosperm development [4]–[11].

Members of the homeotic MADS-box genes encode a family of transcription factors that fulfill the important functions of regulating vegetative growth and flowering time, controlling meristem and floral organ identity, and determining fruit and seed development [12]–[13]. Many MADS-box genes have been identified to constitute an intricate network controlling the orchestration of the floral transition and floral development [14]–[25]. Important key developmental biology questions that remain unanswered include: how is the pattern of formation accomplished, and how does the genetic interaction of floral homeotic genes occurs at the molecular level? Although extensive knowledge on these MADS domain transcription factors that regulate the floral transition and floral organ development is available, little is known regarding the molecular mechanisms they employ to act as the developmental switches for specifying the female reproductive unit in flowering plants. In addition, it is unclear how the homeotic transcription factors organize the spatial patterns of cell differentiation during diverse or specific developmental processes such as embryonic shoot/root initiation and endosperm formation in a developing seed.

The ABC/DE model [13], [18]–[20] of floral development describes the genetic interaction of the five major classes of floral selector genes, and each class determines the identity of different floral organs: sepals, petals, stamens and carpels. The B_s_-group MADS-box genes are close relatives of the B class of the MADS-box gene, a family only described in a few plant species [24], [26]–[33]. In eudicot plants, the B_s_-group MADS-box genes (*e.g.* FBP24 in petunia and *ABS, STK* and *GOA* in *Arabidopsis*) are essential for regulating integument, seed coat, fertilization and endosperm development, and fruit growth [28]–[32]. However, information on B_s_-function genes (*e.g.* rice *MADS29, MADS30* and *MADS31*) in monocots still remains limited [24], [27], [33]. In previous studies, the *FEMALE-STERILE* (*FST*) gene, a B_s_-group MADS-box transcription factor, was reported to putatively control the identity of ovule, pericarp and seed development, as well as the translocation of reserve nutrients during the entire reproductive stage [34], [35]. In addition, the *fst* phenotype is genetically sporophytic and controlled by a single recessive locus in the nucleus [34]. *OsMADS29* (whose ORF is identical to that of *FST*) plays an important role in seed germination [24] and affects the degradation of the nucellus and nucellar projections during the development of a rice seed [33] although this function appears to be restricted to the post-fertilization stage.

In this study, we report that *FST* (DQ004266) acts as positional determinants regulating chalaza formation, integument morphogenesis and the early development of the zygotic embryo and endosperm. *FST* is expressed in reproductive organs throughout all developmental processes. Importantly, the spatio-temporal expression pattern revealed the stepwise formation of a prominent *FST* gene product. This product accumulated in sporophytic organs/tissues in the apical-basal region of ovules and proembryos, as well as the center of endosperm nuclei tissue, pericarp, and seed coat. Microarray and gene ontology (GO) analyses unraveled substantial changes in the expression level of many genes in *fst* mutant ovules and seeds, with a subset of genes involved in developmental and hormonal pathways appearing to be down-regulated. This finding supports a novel function for the B_s_-group MADS-box genes in plants. The discovery of *FST* offers novel insights into developmental biology processes and contributes to a better understanding of the mechanisms regulating the female reproductive unit and seed development mediated by floral homeotic genes in flowering plants.

## Results

### The *fst* Mutation Leads to Sporophytic Female Sterility

**Figure 1:**
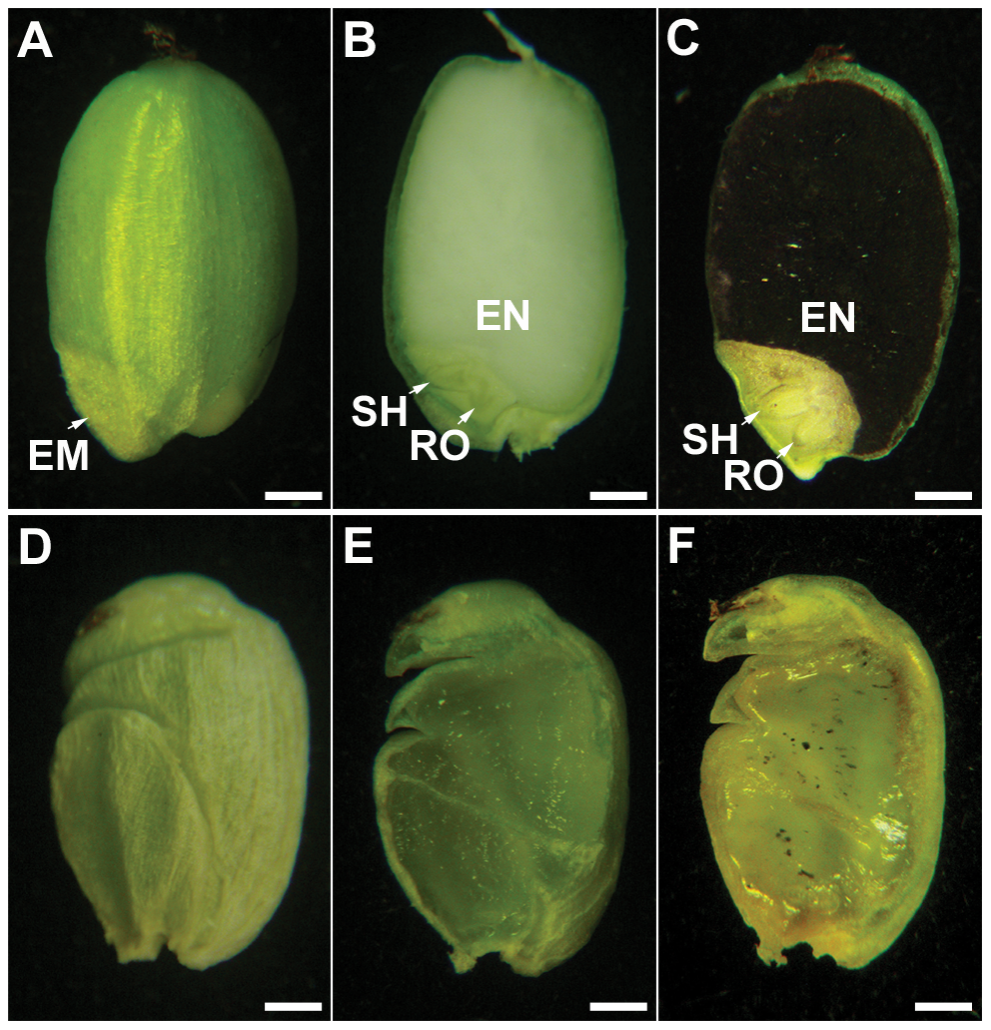
Comparison of the shape and structure of hulled grain in the *fst* mutant and WT. (A–C) The WT plants produced seeds with a normal embryo (EM) and endosperm (EN) 20 DAP. SH, shoot; RO, root. (D–F) The *fst* mutant plants produced pseudo seeds with an abnormal seed coat but no embryo (EM) and endosperm (EN) 20 DAP. Longitudinal sections of mature seeds (B, C, E & F). Starch of endosperm stained with 1% I_2_-KI (C & F). Scale bars, 100 µm.

The spontaneous *fst* mutant plants exhibited similar morphology to that of the wild-type (WT) plants but are completely female sterile. The size, shape and structural composition of floral organs in the *fst* mutant were identical to those of the WT (Figure S1A-S1D). The *fst* mutation did not affect anther development and pollen viability (Figure S1B, S1E & S1F). Pollen from both the *fst* mutant and WT germinated normally in the *fst* mutant style (Figure S1E & S1F), but unlike WT (Figure 1A-1C) the *fst* mutant plants did not produce any visible embryo and endosperm when pollinated with their own pollen or with WT pollen (Figure 1D-1F). All the megagametophytes derived from heterozygous *FST/fst* (+/-) plants were completely fertile. The ratio between fertile and sterile plants was 3:1 in progeny derived from the heterozygous *FST/fst* (+/-) plants (n = 2036). These results confirmed that *fst* is responsible for sporophytic female-specific sterility in rice, which is completely different from other reported female-sterile mutations in rice that are controlled by quantitative traits or two recessive genes [36]–[37].

### The *fst* Mutation Disrupts Development of the Ovule, Zygotic Embryo and Endosperm

**Figure 2:**
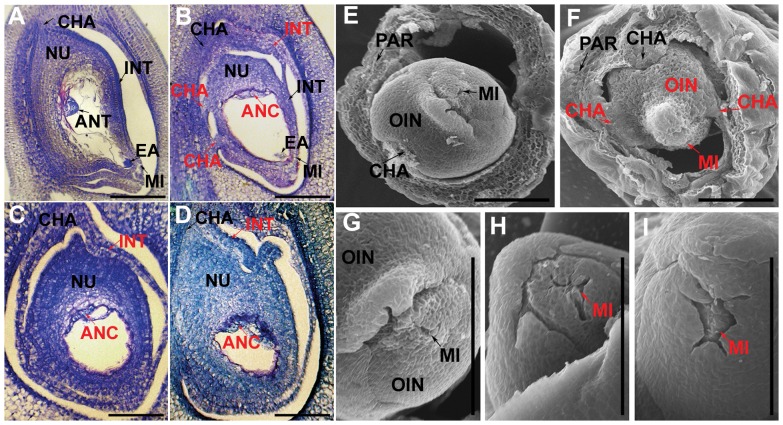
Characterization of ovule and female gametophyte in the *fst* mutant. (A-I) Comparison of ovule development in *FST* (A, E &G) and *fst* (B–D, F, H and I). Embryo sac development completion at the stage just BF (A–F), antipodal cells (ANC) of *fst* were unable to mature as antipodal tissues (ANT) and were located at the correct site within the embryo sac (B), while the ANC of *FST* were fully developed to ANT (A). Polar nuclei (PN) and egg apparatus (EA) in both *FST* and *fst* were developed and appeared normal in shape. At the stage just BF (A–D), abnormalities in mature ovaries showed defects in the development of integuments (INT) and chalaza (CHA) in the *fst* mutant (B–D) compared to the *FST* plants (A). (A–D) was stained with toluidine blue. (E–I) Shape and structure of an *FST* (E & G) and *fst* (F, H & I) mature ovule. Structure of mature ovules observed under SEM (E–I). Arrows: normal structures. OIN, outer integument; PAR, pericarp (seed coat). Red arrows show abnormalities specific to *fst* with defects in antipodal tissues (ANT), chalaza (CHA), integuments (INT) and micropyle (MI). NU, nucellus. Scale bars, 100 µm.

To dissect the cyto-embryological mechanism of sterility in the *fst* mutant, morphological and anatomical analyses were conducted with hundreds of flowers sampled at different stages, ranging from the panicle and floral initiation to ripening during pre- and post-fertilization. Integument development in the *fst* mutant was severely affected and consequently the transformed integuments formed additional chalaza-like (Figure 2B & 2F) and zigzag (Figure 2C & 2D) structures. Similarly, the *fst* micropyle and ovule shape were aberrant (Figure 2H & 2I).

We investigated megasporogenesis and megagametogenesis (data not shown), and did not observe any distinct abnormalities in these developmental processes in the *fst* mutant. After cellularization, however, the shape of the *fst* embryo sac was altered and the antipodal tissues did not mature and position correctly (Figure 2B–2D) when compared to those of the WT (Figure 2A).

**Figure 3:**
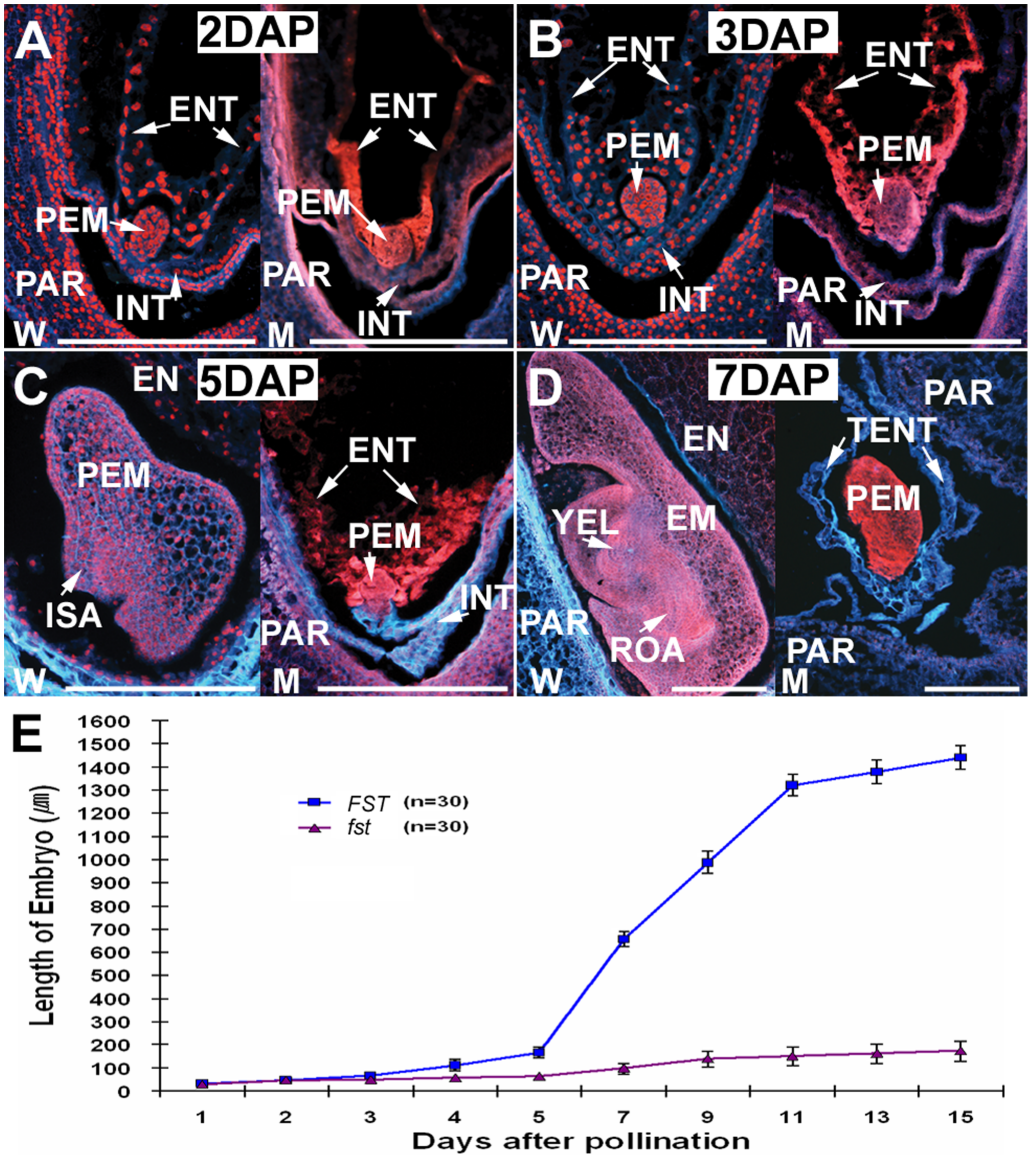
Embryogenesis and endosperm development in the *fst* mutant. (A&B) Proembryo (PEM) and free endosperm tissue (ENT) development of *FST* (W) and *fst* (M) 2 (A) and 3 DAP (B).(C) Growth of the initial shoot apex (ISA) and seminal root primordium differentiation 5 DAP.(D) First leaf primordium differentiation 7 DAP.(E) Developmental changes illustrating embryo length. Arrows: EM, embryo; EN: endosperm; ENT, endosperm nuclei tissues; INT, integument; PAR, pericarp (seed coat); ROA, root apex; TENT, trace of endosperm nuclei tissues; YEL, young embryonic leaves; n, number of embryos measured. Scale bars, 100 µm.

To examine whether the observed morphological defects in ovule development influence fertilization, we examined mature *fst* ovules with and without pollination. Notably, the defective development of integuments and micropyle did not affect fertilizations when *fst* ovules were pollinated with either their own pollen or the WT’s. The early development of both the zygotic embryo and endosperm did not show distinct differences when compared to that of the WT 1–3 days after pollination (DAP) (Figure 3A, 3B & 3E). Nevertheless, the proembryo and endosperm nuclei tissue were arrested at 3–5 DAP and degenerated tissues were detected 5–7 DAP in the *fst* ovules (Figure 3C, 3D & 3E). In contrast, no embryogenesis and endosperm development events were observed in the mutant ovules without pollination (data not shown). Consequently, both morphological and functional defects in the *fst* mutant eventually caused complete failure of embryo and endosperm development after double fertilization.

### Defective Ovule Development and Subsequent Zygote Abortion in the *fst* Mutant are Caused by a Frame-Shift Mutation in the B_s_ MADS-Box Domain

We mapped the *fst* locus using an F_2_ population derived from a hybrid between the rice cultivar Samgangbyeo and the *fst* mutant (spontaneous mutation identified from the rice cultivar Junambyeo; Figure S2A).

Sequence comparison revealed that the genomic DNA of the WT allele *FST* was approximately 4 kb long with 8 exons and 7 introns. Exon 2 carries the ATG initiation codon and contains a highly conserved MADS domain (Figure S2B). The mutant gene *fst* produced a non-functional transcript due to an 8-bp nucleotide deletion, causing a translational frame shift in the MADS-box domain at exon 2 (Figure S2C). Homology analysis of the MADS domain protein revealed that the FST protein is closely related to FBP24 in petunia and ABS in *Arabidopsis* in the B_s_-group MADS-box genes [26]–[27] (Figure S2D & S2E).

**Figure 4:**
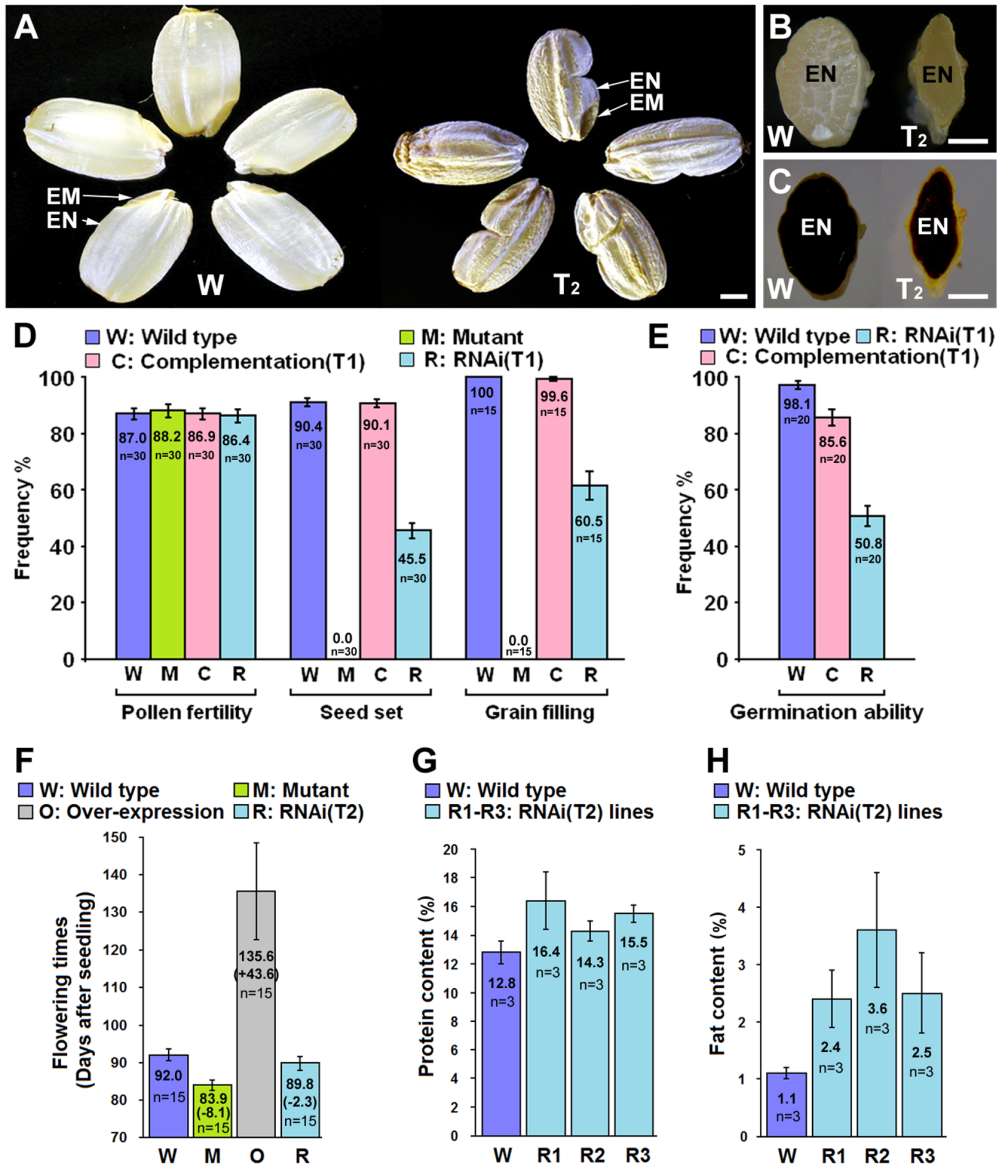
Functional analysis and complementation test of *FST.* (A) Effect of *FST* RNAi (*35S::RNAi-FST*) repression on seed development, showing incomplete grain filling in T_2_ plants. W, WT. (B&C) Comparison of grain filling of the endosperm (EN) (B), starch stained with 1% I_2_-KI (C) in the *FST* (W) and RNAi transgenic seeds at the T_2_ generation. (D) Comparison of pollen fertility, seed set, degree of grain filling in the *FST* (W), *fst* (M) and transgenic plants derived by a complementation test (C) and RNAi silencing (R) of *FST* gene. n, number of individual plants measured. (E) Seed germination frequencies of *FST* (W) and transgenic plants derived by a complementation test (C) and RNAi silencing (R) of *FST* gene. (F) Comparison of flowering time of WT (*FST),* mutant (*fst*) and transgenic plants derived by repression (RNAi) and over-expression of the *FST* gene. Arrows: EM, embryo; EN, endosperm; n, number of individual plants measured. Scale bars, 1 mm. (G) Comaprision of total protein content of WT (*FST)* and transgenic plants derived by repression (RNAi) of the *FST* gene. (H) Comaprision of fat content of WT (*FST)* and transgenic plants derived by repression (RNAi) of the *FST* gene.

To verify the function of the *fst* gene, we performed complementation analysis. Two binary plasmids, carrying either a 1.3-kb full-length cDNA of the WT FST and a 2.6-kb upstream region (*pFST::FSTc*) or a 6.9-kb WT genomic DNA fragment containing the entire ORF plus a 2.6-kb upstream region (*pFST::FSTg*), were incorporated into the genome of the *fst* mutant by an *Agrobacterium*-mediated transgenic approach. Both transgenic plants carrying the individual constructs were able to restore spikelet fertility. Noticeably, *pFST::FSTg* fully restored both embryo and endosperm development (Figure 4D). Most seeds derived from T_0_ and T_1_ transgenic plants germinated with normal shoots and roots (Figure 4E). However, in some of seed recovered by *pFST::FSTc*, the shoot and root germinated on the inner side of the endosperm, causing a reversed hypocotyl axis (Figure S1I & 1J). The T_0_ and T_1_ plants recovered by both *pFST::FSTg* and *pFST::FSTc* showed a high frequency of seed set (>85%) (Figure 4D).

To confirm the function of *FST*, we generated 61 independent transgenic lines with down-regulated *FST* expression by using RNA interference targeting (RNAi) constructs (*35S::RNAi-FST*) in the Nipponbare and Liyu-B backgrounds. Compared to the blank vector transgenic plants, *35S::RNAi-FST* repression plants primarily displayed partial female sterility, defective endosperm development, incomplete grain filling, reduced germination, and increased of total protein and fat contents in rice grain (Figure 4A–4H). In addition, we also created transgenic plants over-expressing *FST* driven by the 35S promoter in Nipponbare and Liyu-B backgrounds. Compared to WT plants, the *fst* and *FST-RNAi* plants flowered 8.1 days and 2.3 days earlier, whereas the *FST* over-expressing plants flowered about 43.6 days later (Figure 4F). The phenotypes resulting from the directed disruption of *FST* expression were similar to the defective developmental phenotypes observed in the *fst* mutant.

### Abundant *FST* Gene Products Accumulate in the Apical-Basal Region of Reproductive Units

**Figure 5:**
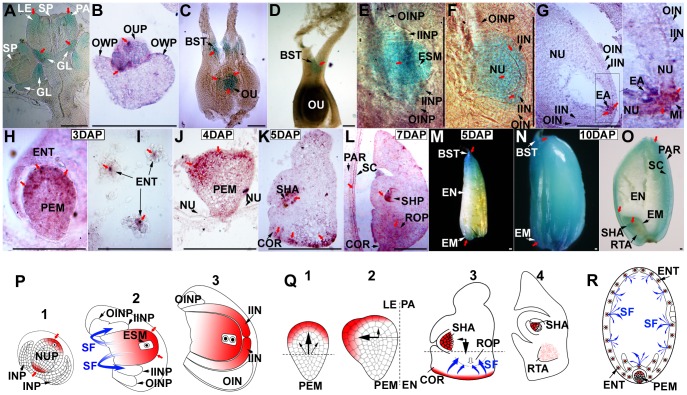
Expression pattern and biological function of *FST.* (A–O) Expression of *FST* during ovule and seed development by *GUS* and *in situ* hybridization. (A–D) *FST* expression in glumes (GL), ovule primordium (OUP), the ovule and the base of stigma (BST) during floral development at the flower primordium stage (A), ovule primordium differentiation stage (B), stigma differentiation stage (C) and stage just BF (D). LE, lemma; OWP, ovary wall primordium; PA, palea; SP, stamen primordia. (E–G) *FST* expression in the nucellus (NU), inner integument (IIN), the tip of nucellus near to egg apparatus (EA) and micropyle (MI) during ovule (OV) and embryo sac (EMS) development at the inner-outer integument differentiation stage (E&F) and stage just BF (G). IINP, inner integument primordium; INP, integument primordium; OIN, outer integument; OINP, outer integument primordium. (H–L) *FST* expression in the apical-basal regions of the proembryo (PEM), endosperm nuclei tissues (ENT), shoot apex (SHA) coleorhiza (COR) and shoot/root primordium (SHP/ROP) of an embryo, and pericarp (PAR) during embryogenesis and endosperm development 3 (H&I), 4 (J), 5 (K) and 7 DAP (L). SC, seed coat. (M–O) *FST* expression at the shoot/root apex (SHA/RTA) of the embryo (EM), endosperm (EN), seed coat (SC) and pericarp (PAR) during seed development 5 (M) and 10 DAP (N&O). (P–R) Models for the expression pattern and biological function of *FST*. Red squares (▪) or arrows (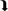) indicate the site of *FST* expression. Blue arrows (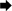) represent the path of the signal factor (SF). During the development of ovules, embryo sacs and seeds, the SFs induced by prominent FST gene products at NUP (P1), EMS (P2&P3), IIN (P3), PEM( Q1&Q2), ISA/COR (Q3&Q4) and ENT (R) could convey positional information for chalaza formation, integument morphogenesis (P2&P3), embryonic patterning (Q1–Q4) and endosperm proliferation (R), respectively. NUP, nucellus primordium. Scale bars, 100 µm.

A fusion protein of FST-GFP driven by the CaMV-35S promoter was expressed exclusively in nuclei of onion cells (Figure S3B). To determine the cellular function of *FST,* we investigated the expression patterns during ovule and seed development using a *β-glucuronidase* (GUS) reporter transgene (*pFST::GUS*). Generally, the *pFST::GUS* construct was expressed in reproductive structures throughout all developmental processes (Figure 5A, 5C–5F & 5M–5O). From the panicle initiation to seed development stages, GUS activity was detected throughout emerging floral primordia and was later confined to the sexual reproductive organs inside the ovary, stamen primordium, tips of lemma, palea, glumes, anther wall, grain apiculus, seed coat and shoot apex of the embryo (Figure 5, Figure S3). In addition, in early pistil development, GUS accumulated at the base of the stigma (Figure 5C & 5D). GUS expression was also observed in the nucellus and inner integument (Figure 5E & 5F). Consistently, both the Northern blot and RT-PCR analyses in samples ranging from seeding to ripening showed no expression of *FST* in vegetative organs such as roots, leaf blades, leaf sheaths, node and internodes of stems. But transcription was detected in reproductive organs such as flowers and seeds during inflorescence and spikelet development (Figure S3F & 3G).

We examined the expression patterns of the *FST* gene in WT plants using *in situ* hybridization. Consistently, the FST transcripts were detected in pistil primordia (Figure 5B), nucellar cells on the micropylar side of the mature ovary (Figure 5G), proembryos 3 DAP (Figure 5H) and free endosperm nuclei 3 DAP (Figure 5I). Interestingly, strong hybridization signals were observed in both the apical and basal parts of the proembryos 3 (Figure 5H) to 4 DAP (Figure 5J), respectively, but these signals were detected exclusively in the shoot apex and coleorhizae in embryos 5 (Figure 5K) and 7 DAP (Figure 5L).

### Comparative Transcriptome Analysis of WT and *fst* Mutant Ovules

We compared the expression profiles of the *fst* mutant and WT at different stages of ovule development using microarray analysis (Affymetrix Rice GeneChip). This allowed us to assess the role of FST in integument morphogenesis, embryogenesis and endosperm development. Three sample replicates were included for both the *fst* mutant and WT to guarantee the reproducibility of microarray analysis.

Before flowering (BF), 59 genes showed at least 4-fold expression level changes in *fst* panicles compared to that of the WT (Table S1) and the 23 main down-regulated genes are displayed at Table 1. GO classification of the down-regulated genes revealed that most genes played important roles in auxin efflux and polarity, gibberellin acid, localization, flower and ovule development, embryonic development, signal transduction, nutrient reservoir, programmed cell death (PCD) and regulation of transcription.

**Table 1:** Genes involved in female organ development with expression changes of at least 4-fold (*fst/* WT) in *fst* floral organs just BF.

Probe Set ID	Fold Change	Average of WBF	Average of MBF	KOMME or Unigene ID	Annotation
Auxin efflux and polarity					
Os.10185.1.S1_at	–15.8	439.79	26.96	AK101049	Similarity to *Xpo5*, *HST*
Os.54026.1.S1_at	–5.3	325.54	61.11	AK101387	Oxidoreductase
Os.24157.1.A1_at	–5.0	133.63	27.17	AK289004	MYCBP-associated protein
GA regulation					
Os.17900.1.S1_s_at	–4.0	4018.62	1010.13	AK110640	*GASA2*
Localization					
OsAffx.30103.4.S1_s_at	–15.3	955.09	61.70	AK103890	*UBQ1, RPL40B*
Flower and ovule development					
Os.1175.1.S1_at	–7.4	151.95	20.30	AK120812	*CK II*
Negative regulation of flowering time					
Os.10185.1.S1_at	–15.8	439.79	26.96	AK101049	Similarity to *Xpo5*, *HST*
Embryonic development					
Os.15983.1.S1_at	–189.0	1462.54	7.55	AK067273	*ATS3*
Signal transduction					
Os.6094.2.A1_s_at	–22.9	206.47	8.91	AK287801	*OsPAD1*
Os.35772.3.S1_at	–19.8	143.94	7.06	AK065867	*RLK17*
Os.50426.1.S1_at	–9.3	69.63	7.16	AK121159	NBS-LRR class
Os.1175.1.S1_at	–7.4	151.95	20.30	AK120812	*OSCKA2*
Os.21562.1.S1_at	–4.0	99.00	22.90	AK242662	*YR5*
Os.20518.1.S1_at	+5.5	40.12	179.58	AK069277	*IP3K*
Nutrient reservoir					
OsAffx.30305.1.S1_at	–115.7	4437.56	20.73	AK242537	LTP family protein
Os.1853.1.S1_at	–4.7	1124.26	262.43	AK062862	*glp16*
Sugar transporter					
Os.10660.1.S1_at	–5.7	340.27	58.23	AK240998	Unknown
Lipid metabolism					
Os.53869.1.S1_at	–6.3	1531.88	255.90	AK100511	Gastric lipase
OsAffx.16962.1.S1_at	–4.3	1031.05	243.80	Os.24116	*CAS1*
Apotosis (programmed cell death)					
Os.26910.1.A1_at	–25.4	155.23	5.93	Os.92013	NBS-LRR class
Os.26510.1.S1_at	–24.6	343.97	13.79	AK066312	CC-NBS class
Os.26992.1.S1_at	–13.7	107.41	7.91	AK100303	*RPM1*
Os.23944.1.A1_at	–13.6	370.75	26.55	Os.24852	*APP*

WBF and MBF: Spikelet was taken from *FST* and the *fst* mutant before flowering stage. Fold change: (–) Down-regulated genes, (+) Up-regulated genes.The definition and function of genes were referenced using SWISS-PROT (by BLASTX) and GO classification (GenBank) in the KOMME (http://cdna01.dna.affrc.go.jp/cDNA/) and Rice Genome Browser (http://rice.plantbiology.msu.edu/cgi-bin/gbrowse/rice/) databases.

Three to five DAP, 406 genes showed at least 4-fold expression changes; moreover, 171 genes displayed at least 10-fold down-regulation in *fst* panicles compared to that of the WT (Table S2). The main representative genes are displayed in Table 2. Genes were functionally grouped into categories involved in auxin efflux and polarity, cell differentiation, embryonic development, seed maturation and endosperm development, morphogenesis, cell-to-cell signaling, signal transduction, nutrient reservoir, starch biosynthesis, sugar and glucose transporter, protein and lipid transport and PCD.

**Table 2:** Genes involved in seed development with expression changes of at least 2-fold (*fst/* WT) in *fst* seeds and ovaries 5 DAP.

Probe Set ID	Fold Change	Average of W5D	Average of M5D	KOMME or Unigene ID	Annotation
**Auxin efflux and polarity**					
Os.26610.1.S1_a_at	–183.9	1741.25	10.48	AK108210	DUF581 domain
Os.8177.1.S1_a_at	–155.7	2313.1	12.81	AK287969	*RP5*
Os.16301.1.S1_at	–122.9	2501.73	34.28	AK288961	*HAP5*
OsAffx.8037.1.S1_s_at	–60.8	1010.47	15.56	AK288062	*ZEAOE17*
Os.10185.1.S1_at	–14.5	112.22	7.49	AK101049	Similarity to *Xpo5*, *HST*
Os.2230.1.S1_at	+2.1	54.74	116.19	AK102343	*PIN1*
**Hormone** **regulation**					
OsAffx.18799.1.S1_s_at	–8.4	386.51	107.63	Os.79618	IAA29-like
Os.12812.1.S1_at	–7.5	295.75	30.29	AK063677	*emp1*
Os.17900.1.S1_s_at	–7.0	98.32	13.3	Os.17900	GA-regulated protein 2
Os.48846.1.S1_at	–6.2	113.89	18.76	AK059073	GA-regulated family protein
**Negative regulation of flowering time**					
Os.10185.1.S1_at	–14.5	112.22	7.49	AK101049	similarity to *Xpo5*, *HST*
OsAffx.16823.1.S1_at	–5.1	311.51	62.10	AK242200	*FIE2*
Os.4171.1.S1_s_at	–4.0	526.58	136.48	AK070121	*OsMADS22*
**Cell differentiation**					
Os.47896.1.A1_at	–24.7	6643.45	269.45	AK107986	Leucine-rich repeat protein
**Cell-cell signaling**					
Os.16615.1.S1_at	–214.8	4896.03	23.23	Os.16615	Protein kinase, putative
Os.28994.1.S1_at	–77.5	2428.35	31.6	AK110796	Hydroxyproline-rich glycoprotein
**Embryonic development**					
Os.5325.1.S1_at	–38.5	256.07	6.15	AK107930	Seed maturation protein
Os.15983.1.S1_at	–24.4	174.06	6.94	AK067273	*AT3*
Os.57554.1.A1_at	–19.9	748.99	66.56	AK241920	NF-YB
**Seed maturation and endosperm development**					
OsAffx.3442.1.S1_at	–5.5	72.34	15.87	AK287592	AIP2
OsAffx.16823.1.S1_at	–5.1	311.51	62.1	AK242200	FIE
Os.39228.1.S1_at	–4.1	153.27	37.29	AK287435	glutathione S-transferase
**Signal transduction**					
Os.2544.1.S1_s_at	–31.4	1960	59.37	AK106383	*OsTIP3*
Os.55500.1.S1_at	–28.7	1012.79	66.03	AK242910	*AFG1*-like protein
Os.47896.1.A1_at	–24.7	6643.45	269.45	AK107986	*LRP*
**Nutrient reservoir**					
Os.13715.3.S1_x_at	–880.0	7177.77	7.32	AK242325	Prolamin/ PPROL 17 precursor
Os.11489.1.S1_a_at	–745.9	8153.94	9.53	AK107343	*CRA1*
Os.8502.7.S1_x_at	–437.2	4046.12	8.92	AK242910	Prolamin 7 gene
Os.5918.1.S1_at	–393.1	3197.39	7.7	AK107785	Prolamin
Os.22346.1.S1_x_at	–343.5	2869.37	7.68	AK288031	*OsGRP1*
Os.9822.3.S1_x_at	–285.5	3657.07	10.83	AK064478	Glutelin type-A 1 precursor
**Starch biosynthesis**					
Os.11244.3.S1_x_at	–90.1	4338.75	114.72	AK109227	Starch synthase
Os.5738.1.S1_at	–21.9	1237.78	55.42	AK106045	Soluble starch synthase II-3
Os.6732.1.S1_a_at	–14.3	2290.43	154.12	AK071497	*APL*
**Apotosis (programmed cell death)**					
Os.26610.1.S1_a_at	–183.9	1741.25	10.48	AK108210	DUF581 domain containing
Os.26910.1.A1_at	–12.0	83.16	6.83	AK073759	*Pikm2-TS*
Os.26510.1.S1_at	–11.9	134.66	11.11	AK066312	*Mla10*

W5D and M5D: Spikelets were sampled from *FST* and the *fst* mutant before flowering stage. Fold change: (-) Down-regulated genes, (+) Up-regulated genes. The definition and function of genes were referenced using SWISS-PROT (by BLASTX) and GO classification (GenBank) in the KOMME (http://cdna01.dna.affrc.go.jp/cDNA/) and Rice Genome Browser (http://rice.plantbiology.msu.edu/cgi-bin /gbrowse/rice/) databases.

Among the genes showing altered expression, *HST* (AK101049), *NF-YB* (AK241920), *ATS3* (AK067237), *FIE2* (AK24220), *LTP* (AK242537), *PPROL17* (AK242325), *CRA1* (AK107343), *OsGRP1* (AK288031), *GASA2* (AK110640), *RPM1* (AK100303) and the *NBS-LRR* family (Os.92013) were involved in polarity specification of the adaxial/abaxial axis, as well as embryonic and seed development [38]–[47].

## Discussion

### A Novel Function Identified in B_s_ MADS-box Genes in Plants

Our results demonstrated that the complete female-specific sterility (*fst*) identified in a *japonica* rice cultivar Junambyeo was caused by a spontaneous mutation in a B_s_ MADS-box gene, although this *fst* mutation does not affect male gamete development and viability (Figure S1A-S1F). Nevertheless, the mutant phenotype of female sterility only occurred in homozygous (-/-) plant, while the female gamete carrying *fst* mutant allele derived from F_1_ heterozygous (+/-) plant was completely fertile, suggesting that the *fst* mutant phenotype of female sterility depended on the sporophytic, instead of gametophytic genotypes. Consistently, morphological and anatomical analyses revealed that, in the *fst* mutant plants, defective developments exclusively occurred in sporophytic (diploid) organs/tissues including integuments, nuclelus cells, zygotic proembryos and endosperm, instead of gametophytic (haploid) embryo sac.

Our data demonstrated that the loss-of-function in the B_s_-group MADS-box gene *FST* influences the patterning and growth of ovules before fertilization but also disrupts early development of the zygotic proembryo and endosperm after the fertilization, leading to subsequent sterility. The severe defect in integument development and transformed ovule shape observed in the *fst* mutant is similar to that observed in the eudicot loss-of-function mutants [28], [29], [30], [32]. Nevertheless, the failure in proembryo development and differentiation leading to aborted seed development is unique in rice *fst* mutant plants. To date, the B_s_-group MADS-box genes were known for regulating integument, seed coat, fertilization, endosperm development, fruit growth, seed germination and degradation of the nucellus and the nucellar projection during rice seed development [24], [26]–[33]. Undoubtedly, rice *FST* plays indispensable roles and multiple functions during ovule and early seed development.

### *FST* Acts as a Negative Regulator of Flowering Time

Our data based on phenotypic analyses demonstrated that the flowering time in plants of the *fst* mutant (loss-of-function in *FST*) and suppressed *FST* (*FST-RNAi*) was significantly earlier than that of WT (*FST*) and over-expressed *FST*. Microarray analysis revealed that expression of *FIE2* (AK242200) and *OsMADS22* (AK070121) was prominently reduced in the *fst* mutant compared to the WT (Table 2). *FIE2*, a polycomb group gene, and *OsMADS22*, a SVP group MADS-box gene, are well-known negative regulators of flowering time [41]–[42]. Thus, we conclude that *FST* may act as a direct or indirect negative regulator of flowering time.

### *FST* Is a Key Regulator Required for Female Organ/Tissue and Seed Development

Our in situ hybridization data showed strong expression of *FST* at the base of the stigma, nucellus and inner integument at early stage of pistil development (Figure 5A–5F). Consistently, morphological and anatomical analyses provided evidence that *fst* mutant plants displayed transformed shape and defective development at the chalaza and integument (Figure 2). This suggests that *FST* may play a pivotal role in regulating development of pistil components. Interestingly, high expression level of *FST* was also detected in apical and basal parts of the 3–4 DAP proembryos, and in the shoot apex and coleorhizae 5–7 DAP embryos (Figure 5H–5L). We therefore postulate the arrest of proembryo development observed in *fst* mutant plants probably resulted from the loss of correctly coding FST protein. In addition, we found that *FST* was expressed in endosperm nuclei tissue and seed coat at an early stage of seed development (Figure 5M–5O). These findings imply that *FST* may participate in regulating endosperm development and nutrient metabolism.

Microarray analyses allowed us to evaluate the impact of *fst* mutation on gene expression, where the level of a large number of genes was significantly altered (Table 1 and 2). Consistent with the results from molecular and morphological analyses, some changes of gene expression appeared to be associated with the developmental defects visualized in *fst* mutant and *FST-RNAi* repression lines. For instance, remarkably down-regulated expression of genes observed in auxin efflux, GA signaling, or other cellular processes, such as PCD, polarity and cell fate determination [38]–[40], [47]–[50], may influence chalaza positioning and integument formation, and subsequently result in defective zygotic embryo and endosperm development.

Based on the observations and analyses, we propose a model to explain how the *FST* works throughout pistil and seed development (Figure 5P–5R) as follows. The expression of *FST* at the base of stigma may define the chalaza formation at correct position by the induction of a signal factor (SF) activated via signal transduction. Failure of this function can cause abnormal shape and positioning of the chalaza in the *fst* mutant. Likewise, the expression of *FST* in the nucellus and inner integument may lead to the development of the nucellus and differentiation of the inner-outer integument by the induction of a SF (Figure 5P2 & 5P3). The high expression of *FST* activates the expression of a subset of genes involved in developmental and hormone signaling pathways that induce cell division in the top section of the proembryo (Figure 5Q1), thus constantly influence morphogenesis and axis determination (Figure 5Q2). In contrast, a low expression level of *FST* may suppress cell division in this process. During differentiation of the primordium into the shoot and root, a high level of *FST* induces differentiation of the shoot apex and determine the axis of shoot and root growth during germination (Figure 5Q3 & 5Q4), respectively. In addition, *FST* may guide the development of ENT (endosperm nuclei tissues) predating ENC (primary cellular endosperm cells) formation and accelerate the building-up of the nutrient reservoir and starch biosynthesis [43]–[46], [51] in the endosperm by the induction of a SF. The phenotype of suppressed *FST* and *OsMADS29* [33] transgenic plants strongly supports this hypothesis.

In conclusion, we demonstrated the multifunctional roles of the B_s_-group MADS-box gene *FST* in rice. Our study provided a complete overview of biological, genetic, and molecular mechanisms, as well as expression profiling data underlying the ovule and seed development. These findings will likely lead to a better understanding of the evolution and molecular mechanisms of reproductive processes in higher plants.

## Materials and Methods

### Mutant Plant History

In 1999, we discovered the spontaneous *fst* mutant G39 (*O. sativa* L. ssp. *japonica*) from Junambyeo, a newly developed rice breeding line in the experimental field of Yeungnam University at Gyeongsan (35.9°N, 128.6°E, 58 masl.) in Korea.

### Experimental Materials

An F_2_ mapping population was derived from a cross between Samgangbyeo (*O. sativa* L. ssp. *indica*) and G39, which contained *fst*. The *fst* allele was introduced into Chinese Dongxiang wild rice (*O. rufipogon* Griff.) and several elite varieties including Junambyeo, Nan34 and Ansanbyeo (*O. sativa* L. ssp. *japonica*) to study the morphology, cytology, inheritance and molecular mechanisms through a backcrossing method.

### Genetic Mapping and Cloning of *FST*

*FST* was mapped with SSR and STS markers using 1286 F_2_ plants. The candidate gene was amplified using gene-specific primers in both *fst* and WT plants (Table S3). The linkage map was constructed with the Map Manager program QTXb17 [52]. ORF search and homology analysis of nucleotide and amino acid sequences were performed using NCBI databases (http://www.ncbi.nlm.nih.gov). The sequence was aligned using MEGA 5.0 [53].

### RNA Isolation and Expression Analysis

Total RNA was prepared using TRI Reagent (Ambion:http://www.ambion. com). First-strand cDNA was synthesized from 2 mg of total RNA. RT–PCR was performed using gene-specific primers (Table S3), 25–30 reaction cycles and three biological replicates for each reaction.

### Microscopic Observations

#### Pollen fertility

Anthers of the *fst* mutant and its WT were collected from 30 spikelets from ten plants (three panicles each) at the flowering stage. Pollen grains from anthers were suspended in a 1% potassium iodide solution (I_2_/KI). Pollen fertility was calculated by determining the percentage of normal pollen grains against total pollen grains per spikelet.

#### Pollination process

Artificial pollination was carried out one day before flowering using previously emasculated mature flowers. Around 3000–3500 perfect flowers from both *fst* and *FST* were emasculated prior to anthesis and enclosed with paper bags. Pollen grains from *fst* were pollinated on its own stigmas and on WT stigmas. Approximately 150–200 pistils (3 replications of 50–70 pistils each) were sampled every 10 min for up to 60 min between 1 and 10 DAP. The pistils were placed in fixative FAA (80% ethanol: 37% formaldehyde: 100% acetic acid, in a ratio of 8:1∶1) for 24 h, rinsed in distilled water for 4 h, softened for 1 h at 60°C in 1N NaOH and rinsed again for 4 h in distilled water. The pistils were then stained in 0.1% water-soluble aniline blue for 10 min. Samples were immediately prepared in a droplet. For each genotype, the ovary was separated from the base of the style during the sample preparation procedure. The samples were then covered with cover slips and squashed gently. The ovules was observed under a microscope (Olympus BX51, UV filter set) and photographed.

#### Ovary development

To analyze ovary development of both the *fst* mutant and its WT, several hundreds of flowers were prepared at different stages ranging from panicle and floral organ initiation to ripening during pre- and post-fertilization. The lemma, palea and anthers were dissected out from the flowers in order to reach the ovaries. The ovaries were immersed in glutaraldehyde fixative solution, which contained 1.4% glutaraldehyde, 2% paraformaldehyde and 50 mM PIPES (pH 7.2), at 4°C overnight as described previously [54]. After rinsing again in PIPES buffer, the samples were dehydrated in an ethanol series of 10 to 100% and then embedded in paraffin (Paraplast Plus, Sigma). The paraffin-embedded ovary samples were further sliced into 4 µm sections with a microtome (Leica DMR) and stained with 0.05% toluidine blue containing 0.1% sodium carbonate or propidium iodide (PI) (5 µm/ml). The tissue sections were observed under a light microscope or in fluorescent mode (Leica DM 2500, fluorescent set).

Scanning electron microscopy (KYKY-EM3200) of whole mature ovules of both *fst* and *FST* was performed as described previously [55]. Development of ovules and embryo sacs of the transgenic plants were detected with mature ovaries; 20 ovaries were prepared from each of the 50 RNAi plants sampled. To investigate starch formation and endosperm shape in the mutant and RNAi lines, spikelets at 10 and 20 DAP were sampled. The endosperms were cut with a sharp knife and stained with 1% potassium iodine solution (I_2_/KI).

The morphological terminologies of rice organs or tissues are described in Table S4. Statistical analysis was performed by Statistix for Windows version 2.0 by Analytical Software (http://www.statistix.com).

### Microarray Analysis

Three independent biological replicates of the *fst* mutant and WT panicle mRNA at different stages of ovule development were used for microarray experiments. Pools of panicles were used to evaluate genes predominantly expressed in the ovary and seed. For microarray analysis, the standard protocol of the Affymetrix GeneChip service was used when setting up the experiment (CapitalBio, http://www.capitalbio.com). To identify differentially expressed genes, the signal ratio of each gene between the WT and the mutant was calculated. The array data sets were named after the genotype (W, WT; M, *fst* or *cynosure*, a previous designation) and ovule stage pool (BF, before flowering stage; 5D, 5 DAP stage). The definition and function of the genes were referenced using SWISS-PROT (by BLASTX) and GO classification (GeneBank) in the KOMME (http://cdna01.dna.affrc.go.jp/cDNA/) and Rice Genome Browser (http://rice.plantbiology.msu.edu/cgi-bin/ gbrowse/rice/) databases.

### Northern Blot Analysis and *In Situ* Hybridization

For Northern blot analysis, 15 µg of purified total RNA from tissues of vegetative and reproductive organs was run on a 1.2% agarose gel containing formaldehyde and transferred onto a Hybond-N+ membrane (Amersham: http://www. gelifesciences.com /Amersham). The gene-specific probe was amplified using the primers *FST*RT-F and *FST*RT-R (Table S3) to amplify a region of ∼200 bp on the 3’ end of the ORF of *FST*. PCR fragments were inserted into the pGEM-T easy vector (Promega: http:/www.promega.com) and transcribed *in vitro* by either a T7 or SP6 promoter for sense or antisense strand synthesis using the Digoxigenin RNA labeling kit (Roche: http://www.roche-applied-science.com). The blot was performed as described previously [56].

For *in situ* hybridization, hybridization and immunological detection were performed as described by Jackson [56] using the same probe generated for the Northern blot analysis.

### Binary Vector Constructs

To determine the complementation of the *FST* phenotype to an 8 bp deletion, *pFST::FSTg* (harboring a 6.9-kb genomic DNA fragment containing the entire *FST* coding region, a 2.6 kb promoter region and a 0.7 kb 3’ region) and *pFST::FSTc* (containing a 2.6 kb promoter region and cDNA sequence of *FST*) were constructed using the binary vector pCAMBIA1300. To construct the *FST* RNAi vector (*35SX2::RNAi-FST*), an intron fragment containing 155 bp was used as a linker between a 396 bp gene-specific fragment in the antisense and sense orientations; these reconstructed fragments were inserted into the pHB binary vector containing a double 35S promoter. A transgenic *35S::FST* over-expressing line of plants was also generated. To study promoter activity, a 2.6 kb genomic DNA fragment upstream of the *FST* coding region was fused to the GUS reporter gene with the nopaline synthase terminator and cloned into the binary vector pCAMBIA1305 to generate the *pFST::GUS* plasmid.

### Plant Transformation

The binary plasmids were introduced into *Agrobacterium tumefaciens* EHA105 and the calli induced from the anther of the *fst* mutant plants were transformed. The binary vectors were also transformed into rice calli, which were induced from mature WT embryos of Nipponbare and Liyu B (*O. sativa* L.). Transgenic plants were selected by hygromycin resistance and subsequently transferred to soil. In total, 17, 12, 61 and 30 independent transgenic lines were obtained for the *pFST::FSTc*, *pFST::FSTg*, *35SX2::RNAi-FST* and *pFST::GUS* constructs, respectively. All transgenic materials were assayed in the T_0_ and/or T_1 ,_ T_2_ generations using 10 to 30 independent or sibling plants.

For the nuclear localization analysis, the binary plasmid *35S::FST-GFP* was transformed into onion epidermal cells. The expression of the fusion protein was observed with a fluorescent microscope.

### Biological Trait and Physiochemical Property Analysis

For all samples of the mutant, WT and transgenic lines, the degree of grain filling was measured by 100 seed-weight from 15 individual plants (100 seeds each) while the WT was used as the control. In addition, the germination ability was evaluated using 30 individual plants (100 seeds each) and the seeds were germinated for 3 days at 28°C. The total protein and fat contents of grain were detected by using NIR spectra (Bruker FT-NIR, Vector 22/N-I) with three biological replicates from three independent RNAi lines (three plants per line) and WT plants.

### Promoter Activity Detection

For the GUS assay, transgenic plants were harvested from various tissues at different developmental stages and fixed in a solution of 2% paraformaldehyde, 1 mM EDTA, 100 mM sodium phosphate buffer pH 7.0. GUS activity was analyzed by staining overnight at 37°C in a staining solution (0.5% Triton X-100, 2 mM X-GluA, 50 mM sodium phosphate buffer, pH 7.0).

### Sequencing Data

Sequence data from this article can be found in the NCBI/GenBank data libraries under the accession number DQ004266. The microarray datasets have been deposited in GEO database with the accession number GSE33441.

## Supporting Information

Figure S1 **Phenotypic characterization of the** ***FST*** **and** ***fst*** **plants.** (A&B) Floral organs of *FST* (A) and *fst* (B) at the flowering stage. AN, anther; FI, filament; LE, lemma; LO, lodicules; OV; ovary; PA, palea; ST, stigma. (C&D) Normal seed of *FST* (C) and pseudo-like seed of *fst* (D) at the harvesting stage. (E&F) Pollen (PO) tube (PT) growth of *fst* 30 min after pollination visualized with aniline blue staining (E) and with SEM (F). (G&H) Normal penetration of *fst* pollen tube into the ovule (OU) through the micropyle (MI) at 40 min after pollination with SEM (G) and with aniline blue staining (H). TIPT, tip of pollen tube; TRPT, trace of pollen tube; OIN, outer integument. (I) Seed recovered by *pFST::FSTc*; shoot and root germinating on the inner side of endosperm caused a reversed hypocotyl growth axis. (J) Model of the structure and germination of rice seeds. EN, endosperm; LE, Lemma; PA, Palea; PAR, Pericarp; RO, Root; RTA, Root apex; SH, Shoot; SHA, Shoot apex; VBS, Vascular bundle of scutellum. Scale bars, 100 µm.
(TIF)

Figure S2 **Molecular mapping, cloning and phylogenetic analysis of the** ***FST*** **gene.** (A) Fine mapping of *fst* on chromosome 2. (B) Diagram of *FST* within a 16-kb region on the YAC clone AP 4836. Black boxes, exons; thin lines, introns; horizontal arrow, direction of transcription; white triangle, deleted *fst* region. (C) Schematic representation of *FST* and deletion in the MADS-box domain of *FST*. ORFs are boxed and regions therein are indicated. Black arrows, 8-bp nucleotide deletion shown in red. (D) Phylogenetic tree based on a comparison of the full amino acid sequences of typical or representative MADS-box genes in rice and other plants. Shown is a simplified cladogram illustrating the consensus most-parsimonious pattern of the relationships obtained using MEGA. The names of the MADS-box genes are indicated based on previous reports. (E) Alternative splicing and alignment of *FST* BF and *FSTb* 3–5 DAP, respectively.
(TIF)

Figure S3 **Subcellular localization and expression of the** ***FST*** **gene.** (A & C–E) GUS accumulation of pFST::GUS at the stages of panicle differentiation (PDS) (A), early stamen and pistil organ development (early meiosis to late meiosis) (SPD) (B), 5 DBF to just BF (BPS) (C), ripening (D) and 10 DAP (E). GUS accumulated in the lemma (LE), palea (PA), stamen primordia (SP), base of the stigma (BST), ovule (OV), tip of anther (AN) walls, apiculus (API), glumes (GL) of flowers and new tiller bud (TB) at the base of the main culm (BMC). (B) Nuclear localization of *FST* in onion epidermal cells transformed with the *35S::FST-GFP* vector. *FST-GFP* was observed in nuclei. (F) *FST* expression pattern in vegetative organs (root, leaf blade, leaf sheath, node and internode of stem) and reproductive organs (panicles) determined by RT-PCR. *FST* showed low expression at the SPD stage but high expression at the pollination and fertilization stages (30–60 min after pollination) (PFS). SES, seedling stage; TIS, tillering stage; PDS, panicle differentiation stage (panicle length < 5 mm); SPD, stamen and pistil organ development stage (panicle length 5–10 cm, early meiosis to late meiosis). (G) *FST* expression pattern in panicles by Northern blot analysis. Scale bars, 25 µm.
(TIF)

Table S1 **Genes involved in female organ development BF with expression changes of at least 4-fold (** ***FST/fst*** **).**
(XLS)

Table S2 **Genes involved in seed development 5 DAP with expression changes of at least 10-fold (** ***FST/fst*** **).**
(XLS)

Table S3 **Primers used for genotyping, plasmid construction and gene analyses in this study.**
(PDF)

Table S4 **Abbreviations of the terminologies of rice organs or tissues used in the paper.**
(PDF)
