# Supplementary figures and images for: The Bsister MADS Gene FST Determines Ovule Patterning and Development of the Zygotic Embryo and Endosperm
Source: PLoS One. 2013 Mar 19;8(3):e58748. doi: 10.1371/journal.pone.0058748 (PMC3602522; doi:10.1371/journal.pone.0058748)

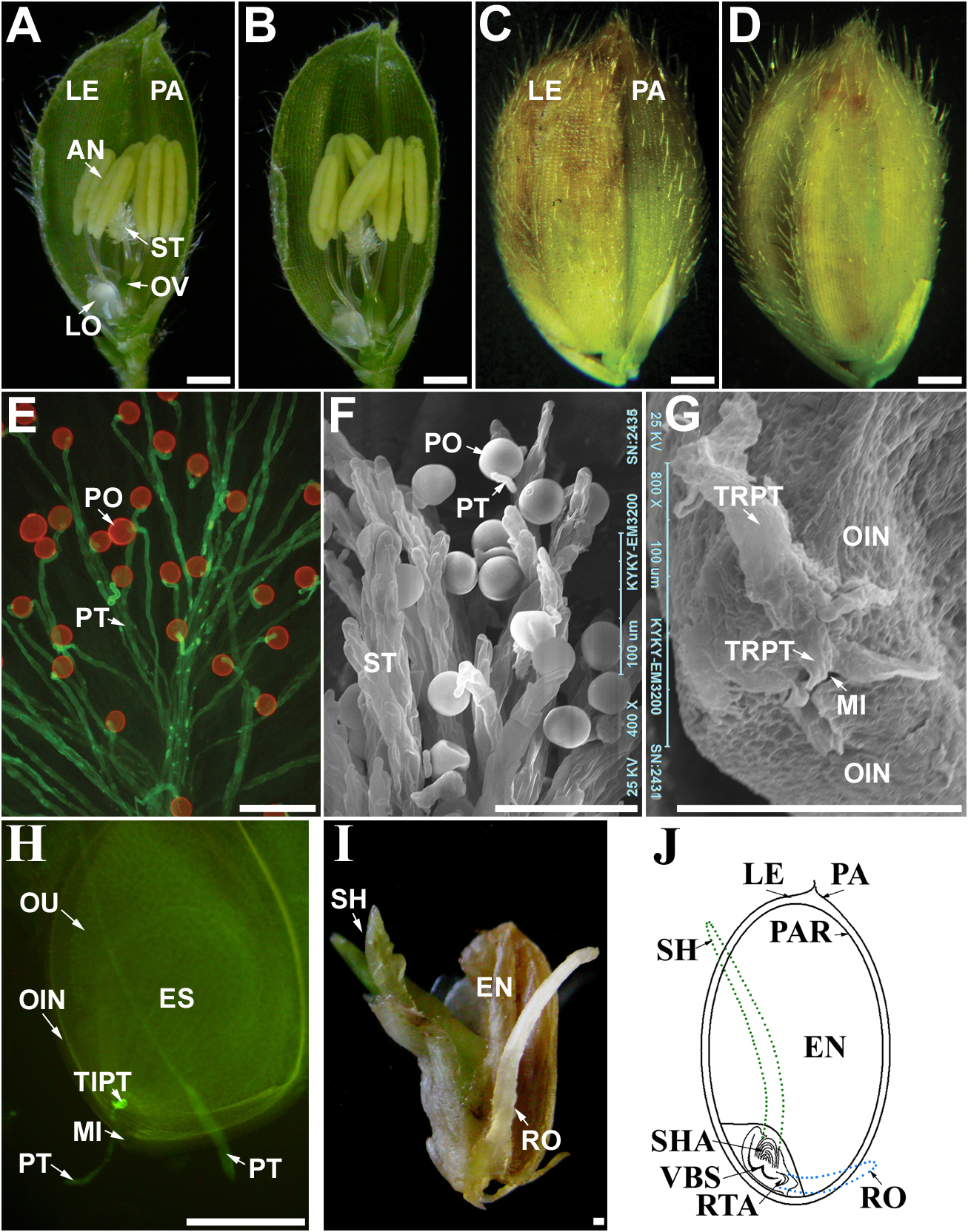

Supplement: Figure S1 — Phenotypic characterization of the FST and fst plants. (A&B) Floral organs of FST (A) and fst (B) at the flowering stage. AN, anther; FI, filament; LE, lemma; LO, lodicules; OV; ovary; PA, palea; ST, stigma. (C&D) Normal seed of FST (C) and pseudo-like seed of fst (D) at the harvesting stage. (E&F) Pollen (PO) tube (PT) growth of fst 30 min after pollination visualized with aniline blue staining (E) and with SEM (F). (G&H) Normal penetration of fst pollen tube into the ovule (OU) through the micropyle (MI) at 40 min after pollination with SEM (G) and with aniline blue staining (H). TIPT, tip of pollen tube; TRPT, trace of pollen tube; OIN, outer integument. (I) Seed recovered by pFST::FSTc; shoot and root germinating on the inner side of endosperm caused a reversed hypocotyl growth axis. (J) Model of the structure and germination of rice seeds. EN, endosperm; LE, Lemma; PA, Palea; PAR, Pericarp; RO, Root; RTA, Root apex; SH, Shoot; SHA, Shoot apex; VBS, Vascular bundle of scutellum. Scale bars, 100 µm. (TIF) [file pone.0058748.s001.tif]

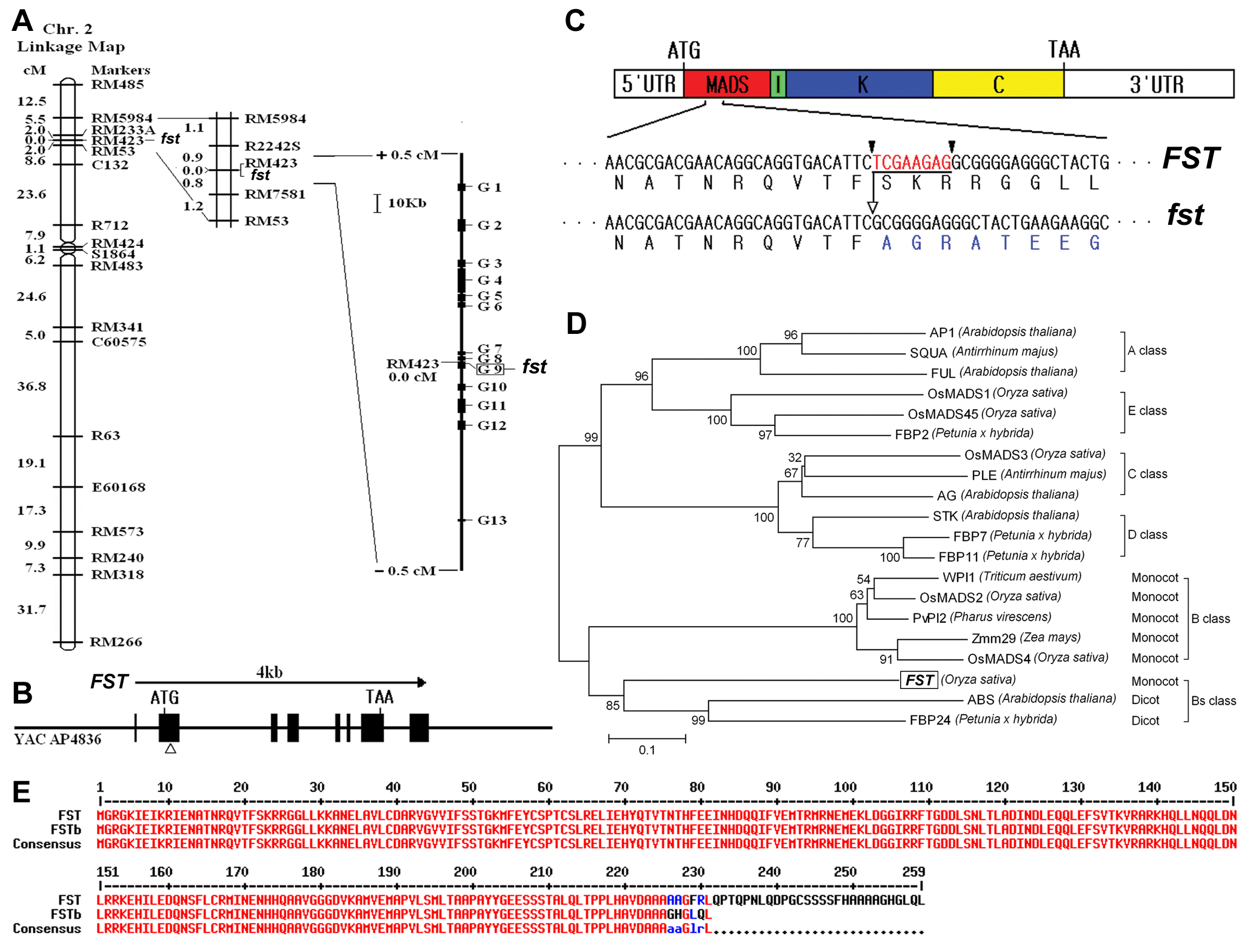

Supplement: Figure S2 — Molecular mapping, cloning and phylogenetic analysis of the FST gene. (A) Fine mapping of fst on chromosome 2. (B) Diagram of FST within a 16-kb region on the YAC clone AP 4836. Black boxes, exons; thin lines, introns; horizontal arrow, direction of transcription; white triangle, deleted fst region. (C) Schematic representation of FST and deletion in the MADS-box domain of FST. ORFs are boxed and regions therein are indicated. Black arrows, 8-bp nucleotide deletion shown in red. (D) Phylogenetic tree based on a comparison of the full amino acid sequences of typical or representative MADS-box genes in rice and other plants. Shown is a simplified cladogram illustrating the consensus most-parsimonious pattern of the relationships obtained using MEGA. The names of the MADS-box genes are indicated based on previous reports. (E) Alternative splicing and alignment of FST BF and FSTb 3–5 DAP, respectively. (TIF) [file pone.0058748.s002.tif]

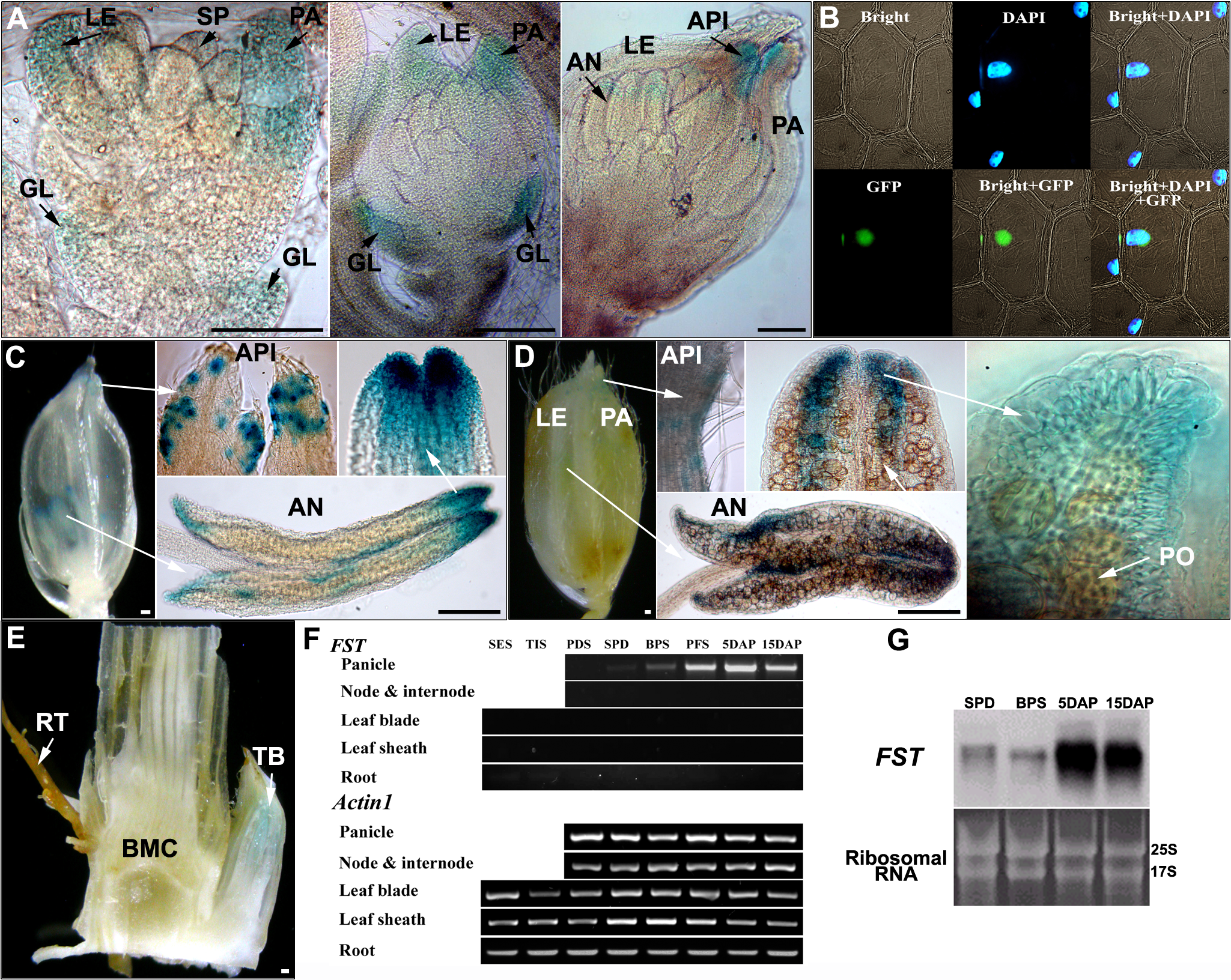

Supplement: Figure S3 — Subcellular localization and expression of the FST gene. (A & C–E) GUS accumulation of pFST::GUS at the stages of panicle differentiation (PDS) (A), early stamen and pistil organ development (early meiosis to late meiosis) (SPD) (B), 5 DBF to just BF (BPS) (C), ripening (D) and 10 DAP (E). GUS accumulated in the lemma (LE), palea (PA), stamen primordia (SP), base of the stigma (BST), ovule (OV), tip of anther (AN) walls, apiculus (API), glumes (GL) of flowers and new tiller bud (TB) at the base of the main culm (BMC). (B) Nuclear localization of FST in onion epidermal cells transformed with the 35S::FST-GFP vector. FST-GFP was observed in nuclei. (F) FST expression pattern in vegetative organs (root, leaf blade, leaf sheath, node and internode of stem) and reproductive organs (panicles) determined by RT-PCR. FST showed low expression at the SPD stage but high expression at the pollination and fertilization stages (30–60 min after pollination) (PFS). SES, seedling stage; TIS, tillering stage; PDS, panicle differentiation stage (panicle length < 5 mm); SPD, stamen and pistil organ development stage (panicle length 5–10 cm, early meiosis to late meiosis). (G) FST expression pattern in panicles by Northern blot analysis. Scale bars, 25 µm. (TIF) [file pone.0058748.s003.tif]
